# Impact of Permissive Hypoxia and Hyperoxia Avoidance on Clinical Outcomes in Septic Patients Receiving Mechanical Ventilation: A Retrospective Single-Center Study

**DOI:** 10.1155/2021/7332027

**Published:** 2021-10-14

**Authors:** Kota Nishimoto, Takeshi Umegaki, Sayaka Ohira, Takehiro Soeda, Natsuki Anada, Takeo Uba, Tomohiro Shoji, Munenori Kusunoki, Yasufumi Nakajima, Takahiko Kamibayashi

**Affiliations:** Department of Anesthesiology, Kansai Medical University Hospital, 2-3-1 Shin-machi, Hirakata, Osaka 573-1191, Japan

## Abstract

**Background:**

Septic patients often require mechanical ventilation due to respiratory dysfunction, and effective ventilatory strategies can improve survival. The effects of the combination of permissive hypoxia and hyperoxia avoidance for managing mechanically ventilated patients are unknown. This study examines these effects on outcomes in mechanically ventilated septic patients.

**Methods:**

In a retrospective before-and-after study, we examined adult septic patients (aged ≥18 years) requiring mechanical ventilation at a university hospital. On April 1, 2017, our mechanical ventilation policy changed from a conventional oxygenation target (SpO_2_: ≥96%) to more conservative targets with permissive hypoxia (SpO_2_: 88-92% or PaO_2_: 60 mmHg) and hyperoxia avoidance (reduced oxygenation for PaO_2_ > 110 mmHg). Patients were divided into a prechange group (April 2015 to March 2017; *n* = 83) and a postchange group (April 2017 to March 2019; *n* = 130). Data were extracted from clinical records and insurance claims. Using a multiple logistic regression model, we examined the association of the postchange group (permissive hypoxia and hyperoxia avoidance) with intensive care unit (ICU) mortality after adjusting for variables such as Sequential Organ Failure Assessment (SOFA) score and PaO_2_/FiO_2_ ratios.

**Results:**

The postchange group did not have significantly lower adjusted ICU mortality (0.67, 0.33-1.43; *P* = 0.31) relative to the prechange group. However, there were significant intergroup differences in mechanical ventilation duration (prechange: 11.0 days, postchange: 7.0 days; *P* = 0.01) and ICU stay (prechange: 11.0 days, postchange: 9.0 days; *P* = 0.02).

**Conclusions:**

Permissive hypoxia and hyperoxia avoidance had no significant association with reduced ICU mortality in mechanically ventilated septic patients. However, this approach was significantly associated with shorter mechanical ventilation duration and ICU stay, which can improve patient turnover and ventilator access.

## 1. Introduction

Patients with sepsis often require mechanical ventilation for respiratory dysfunction, and the use of different ventilatory strategies can affect survival rates [1]. As mortality rates have been reported to exceed 30% in mechanically ventilated septic patients [2, 3], the improvement of mechanical ventilation protocols may have important implications for survivability.

A randomized controlled trial is currently examining the effects of permissive hypoxia in mechanically ventilated patients with respiratory dysfunction [4], and a recent review indicated that permissive hypoxia may provide a better risk-benefit profile than the restoration of normoxia [5]. The dissociation curve of arterial oxygen tension (PaO_2_) and oxyhemoglobin saturation (SpO_2_) has been shown to be relatively flat when the latter exceeds 90% [6], which supports the rationale of using permissive hypoxia. In contrast, conventional oxygenation targets have been reported to help prevent severe hypoxia [7]. Hypoxia may impair intracellular calcium transport, which in turn reduces the relaxation abilities of the left and right ventricles [8]. In addition, renal tissue hypoxia potentially increases the risk of pulmonary congestion, pulmonary hypertension, and right ventricular overload affecting left ventricular filling [9]. At the molecular level, hypoxia can trigger the expression of hypoxia-inducible transcription factor-1, which suppresses mitochondrial activity and limits the production of reactive oxygen species [10]. As reactive oxygen species elicit the oxidative stress response and induce cell death, their regulation may affect the preservation of organ function [11]. In this way, the clinical benefits of permissive hypoxia gained wide acceptance despite risks of excessive hypoxia. Furthermore, hyperoxia may have detrimental effects on survival among patients in critical condition [12, 13]. Therefore, mechanical ventilation strategies that focus on the optimal range of oxygenation remain an active topic of research. Therefore, a combined oxygenation strategy involving permissive hypoxemia and hyperoxia avoidance may improve the prognosis of mechanically ventilated patients with sepsis. However, there is a lack of evidence on the clinical effects of this strategy.

Until March 31, 2017, our hospital's ventilation policy was to attain an SpO_2_ target of ≥96%. On April 1, 2017, this policy changed to an SpO_2_ target of 88–92% or a PaO_2_ target of 60 mmHg; in cases where PaO_2_ exceeds 110 mmHg, the fraction of inspiratory oxygen (FiO_2_) or peak inspiratory pressure (PIP) settings are reduced. In this study, we hypothesized that this policy shift to the combination of permissive hypoxia and hyperoxia avoidance would improve clinical outcomes in adult septic patients requiring mechanical ventilation. To test this hypothesis, we conducted a retrospective study to compare mortality in patients before and after this policy change.

## 2. Materials and Methods

### 2.1. Study Design and Data Source

We conducted a retrospective single-center analysis of mechanically ventilated septic patients who had been admitted to the general intensive care unit (ICU) of a university hospital in Japan between April 1, 2015, and March 31, 2019. This ICU is a closed unit managed by full-time intensivists. All data were extracted from the medical records and administrative claims of Kansai Medical University Hospital, Japan. This study was conducted in accordance with the principles of the Declaration of Helsinki and was approved by the institutional review board of Kansai Medical University Hospital (Approval Number: 2019030). As the database comprised retrospective claims information, the ethics committee waived the need for written informed consent. The data were not anonymized before being received by the authors and were accessed between April 2019 and November 2020.

### 2.2. Patient Selection

We first identified hospitalized patients aged 18 years or older who had been diagnosed with sepsis and had undergone mechanical ventilation during the study period. In accordance with Sepsis-3 criteria [14], sepsis was defined as a life-threatening organ dysfunction caused by a dysregulated host response to infection leading to an increased Sequential Organ Failure Assessment (SOFA) score of 2 points or more. Although our study patients included some who were treated before the Sepsis-3 criteria were published in 2016 [14], all patients were retrospectively identified based on these criteria. As our hospital's mechanical ventilation policy changed on April 1, 2017, the patients were divided into 2 groups: the prechange group comprised patients who were admitted to the ICU between April 1, 2015, and March 31, 2017, and the postchange group comprised patients who were admitted to the ICU between April 1, 2017, and March 31, 2019. The prechange group received mechanical ventilation with conventional oxygenation targets (SpO_2_ target of ≥96%), whereas the postchange group received mechanical ventilation with permissive hypoxia and hyperoxia avoidance (SpO_2_ target of 88–92% or PaO_2_ target of 60 mmHg; reduction of FiO_2_ or PIP if PaO_2_ > 110 mmHg).

### 2.3. Patient Characteristics

We collected information on the following baseline characteristics: age, sex, height, body weight, source of sepsis (lung, abdomen, urinary tract, soft tissue, mediastinum, endocardium, and others), acute respiratory distress syndrome (ARDS) severity based on the Berlin definition [15], and SOFA score. Body mass index was calculated using the formula body weight (kg) × height^−2^ (m).

All patients in both groups were initially ventilated with airway pressure release ventilation (APRV). The initial mechanical ventilation settings were FiO_2_ of 0.40, PIP of 20–25 cmH_2_O, low expiratory pressure of 0 cmH_2_O, low expiratory pressure duration of 0.5 seconds, and respiratory rate of 10–12 breaths/minute. In the prechange group, the mechanical ventilation settings were adjusted at the discretion of the attending physician. In contrast, the settings in the postchange group were determined based on the new ventilation policy.

Data were also obtained on PIP and plateau pressure just after tracheal intubation, the maximum PIP within 48 hours of tracheal intubation, and the plateau pressure at the maximum PIP within 48 hours of tracheal intubation. We also collected data on PaO_2_ divided by FiO_2_ (P/F ratio) just before and after tracheal intubation and at the maximum PIP within 48 hours of tracheal intubation. Patient-level data on PaO_2_ levels were obtained for up to 1 week after intubation.

### 2.4. Outcome Measures

The primary outcome measure was ICU mortality. The secondary outcome measures were in-hospital mortality, duration of mechanical ventilation, incidence of barotrauma, duration of ICU stay, and duration of hospital stay. Barotrauma included pneumothorax, subcutaneous emphysema, and mediastinal emphysema.

### 2.5. Statistical Analysis

Continuous variables were calculated as means and standard deviations, and categorical variables were calculated as percentages or scores with interquartile ranges (IQRs). To compare the differences between the prechange and postchange groups, we used Student's *t*-test or Welch's *t*-test with Levene's test for continuous variables and the chi-squared test or Fisher's exact test for categorical variables. The various SOFA scores (respiratory, cardiovascular, liver, renal, coagulation, and neurologic) were calculated as median values accompanied by the interquartile range and were analyzed using the Mann–Whitney *U* test. The Shapiro-Wilk test was used to check for normality in the outcome measures with continuous variables; based on the results of this test, we used Student's *t*-test, Welch's *t*-test, or the Mann–Whitney *U* test, as appropriate. A two-way repeated measures analysis of variance was performed to compare the transitions in PaO_2_ levels just after tracheal intubation and at 6-hour intervals thereafter for 1 week.

Multivariate logistic regression analyses were conducted to examine the association between the ventilation policy change and ICU mortality. First, we constructed a logistic regression model with ICU mortality as the dependent variable and the postchange group (ref: prechange group) as the main independent variable of interest; the covariates included patient characteristics and P/F ratios just before tracheal intubation. Next, we conducted propensity score matching (propensity to be assigned to the postchange group) based on SOFA scores and reperformed the regression analysis using the propensity score-matched patients; the covariates included patient characteristics and P/F ratios just before tracheal intubation. Patients were matched using the 1 : 1 nearest neighbor matching within a caliper width of 0.25 standard deviations of the propensity score logit. The regression analysis was also performed using ARDS patients only. The odds ratios (ORs) and 95% confidence intervals (CIs) for the independent variables were calculated.

Kaplan–Meier survival curves were plotted to examine the intergroup differences in survival and time to weaning from mechanical ventilation. We also plotted survival curves for time to weaning from mechanical ventilation according to 3 categories of P/F ratios (≤100, 100.1–200, and >200) just before tracheal intubation.

Two-tailed *P* values lower than 0.05 were regarded as statistically significant. All analyses were performed using SPSS Version 26.0 (IBM Japan, Ltd., Tokyo, Japan).

## 3. Results

### 3.1. Patient Characteristics

The prechange group and the postchange group comprised 83 patients and 130 patients, respectively (Table 1). In this closed ICU, ventilator management and blood gas analyses for all patients were overseen by intensivists. There were no cases in which treatment deviated from the mechanical ventilation policies. There were 43 patients with ARDS (mild: 5, moderate: 11, and severe: 27). We found no significant intergroup differences in patient characteristics, with the exception of SOFA score (*P* = 0.03).  Table 2 shows the characteristics of the propensity score-matched patients (71 patients in each group). There were no significant differences in patient characteristics between the groups.

### 3.2. Mechanical Ventilation Measurements

**Figure 1:**
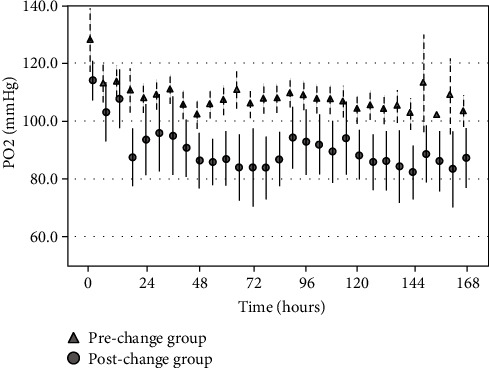
Time course of PaO_2_ measurements. There was a statistically significant difference in the transition of PaO_2_ levels between the prechange and postchange groups (two-way repeated measures analysis of variance: *P* < 0.01). PaO_2_: arterial oxygen tension.

Table 3 shows the PIP levels, plateau pressure levels, and P/F ratios in the prechange and postchange groups. None of the outcome measures exhibited a normal distribution with the Shapiro-Wilk test. Therefore, the Mann–Whitney test was used to compare these outcomes. The postchange group, when compared with the prechange group, showed a significant reduction in PIP just after tracheal intubation (20.0 [IQR: 20.0–25.0] cmH_2_O vs. 25.0 [IQR: 20.0–27.0] cmH_2_O, *P* < 0.001) and maximum PIP within 48 hours (23.0 [IQR: 20.0–25.0] cmH_2_O vs. 25.0 [IQR: 25.0–30.0] cmH_2_O, *P* < 0.001). There were no significant intergroup differences in the P/F ratio just before tracheal intubation (*P* = 0.19), P/F ratio just after tracheal intubation (*P* = 0.58), and P/F ratio at maximum PIP within 48 hours (*P* = 0.38).  Figure 1 presents the time course of PaO_2_ levels just after tracheal intubation and at 6-hour intervals in the prechange and postchange groups. The results showed that the postchange group had significantly lower PaO_2_ levels than the prechange group. These levels were generally maintained within the 60–100 mmHg range in the postchange group (18 hours after intubation), but were kept above 100 mmHg in the prechange group. Furthermore, there was no significant intergroup difference in barotrauma incidence (*P* = 0.91).

After propensity score matching, the postchange group, when compared with the prechange group, showed a significant reduction in PIP just after tracheal intubation (20.0 [IQR: 18.0–25.0] cmH_2_O vs. 25.0 [IQR: 20.0–26.0] cmH_2_O, *P* < 0.001) and maximum PIP within 48 hours (22.0 [IQR:20.0–25.0] cmH_2_O vs. 25.0 [IQR:24.0–30.0] cmH_2_O, *P* < 0.001). There were no significant differences in the P/F ratio just before tracheal intubation (*P* = 0.24), P/F ratio just after tracheal intubation (*P* = 0.84), and maximum PIP within 48 hours (*P* = 0.27).

### 3.3. ICU Mortality and In-Hospital Mortality

There was no statistically significant difference in ICU mortality between the prechange and postchange groups (31.3% vs. 23.1%, *P* = 0.18) (Table 3). In addition, the in-hospital mortality rate was identical in both groups (both 44.6%, *P* = 1.00).

**Figure 2:**
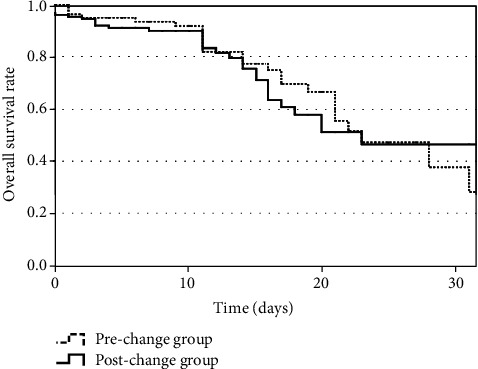
Kaplan–Meier ICU survival curves. There was no significant difference in survival between the prechange and postchange groups (log-rank test: *P* = 0.92). ICU: intensive care unit.

The association between the policy change and ICU mortality was analyzed using a multivariate logistic regression model that adjusted for SOFA score and P/F ratio just before tracheal intubation. As shown in Table 4, the postchange group was not significantly associated with reductions in unadjusted ICU mortality (OR: 0.70; 95% CI: 0.22–2.23; *P* = 0.55) or adjusted ICU mortality (OR: 0.67; 95% CI: 0.33–1.43; *P* = 0.31). A similar lack of association was observed in the propensity score-matched analysis (Table 5) and subgroup analysis of ARDS patients (Table 6). The Kaplan–Meier curves of ICU mortality for the prechange and postchange groups are shown in Figure 2. The postchange group did not have significantly better survival than the prechange group (log-rank test: *P* = 0.92).

### 3.4. Duration of Mechanical Ventilation

**Figure 3:**
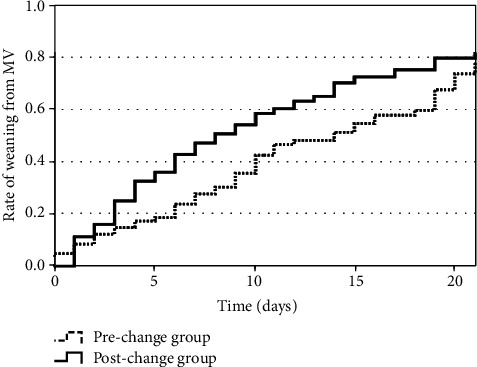
Kaplan–Meier curves for time to weaning from mechanical ventilation. Patients in the postchange group had significantly shorter time to weaning from mechanical ventilation than patients in the prechange group (log-rank test: *P* = 0.02). MV: mechanical ventilation.

**Figure 4:**
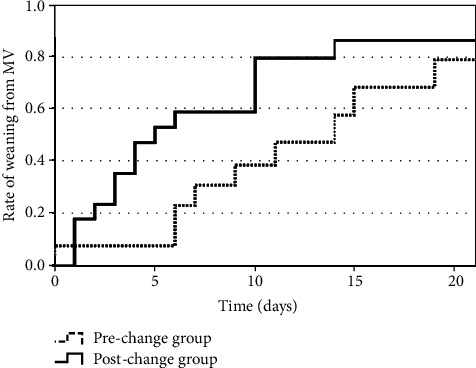
Kaplan–Meier curves for time to weaning from mechanical ventilation in patients with P/F ratios > 200. P/F ratios were taken just before tracheal intubation. There was no significant difference in time to weaning from mechanical ventilation between the prechange and postchange groups (log-rank test: *P* = 0.12). MV: mechanical ventilation.

**Figure 5:**
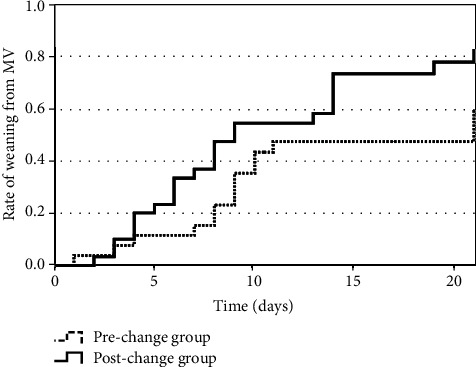
Kaplan–Meier curves for time to weaning from mechanical ventilation in patients with P/F ratios of 100.1–200. P/F ratios were taken just before tracheal intubation. Patients in the postchange group had significantly shorter time to weaning from mechanical ventilation than patients in the prechange group (log-rank test: *P* = 0.04). MV: mechanical ventilation.

**Figure 6:**
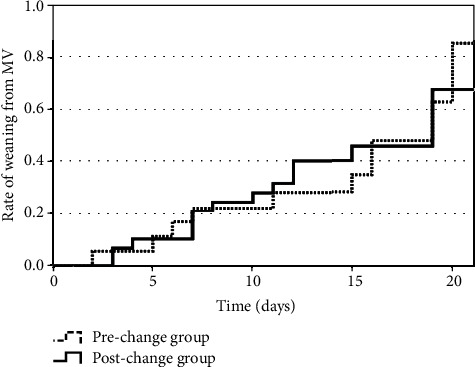
Kaplan–Meier curves for time to weaning from mechanical ventilation in patients with P/F ratios ≤ 100. P/F ratios were taken just before tracheal intubation. There was no significant difference in time to weaning from mechanical ventilation between the prechange and postchange groups (log-rank test: *P* = 0.98). MV: mechanical ventilation.

As shown in Table 3, there was a significant difference in mechanical ventilation duration between the prechange and postchange groups (11.0 [IQR: 6.0–19.0] days vs. 7.0 [IQR: 3.0–14.0] days, *P* = 0.01). The Kaplan–Meier curves of time to weaning from mechanical ventilation are shown in Figure 3. Patients in the postchange group had a significantly shorter time to weaning from mechanical ventilation than those in the prechange group (log-rank test: *P* = 0.02). When stratified according to the P/F ratios just before tracheal intubation (Figures 4–6), we detected a significant intergroup difference in time to weaning from mechanical ventilation among patients with P/F ratios of 100.1–200 (Figure 5); however, there were no significant differences in patients with P/F ratios > 200 and ≤100 (Figures 4 and 6).

### 3.5. ICU Stay and Hospital Stay

As shown in Table 3, there was a significant difference in ICU stay between the prechange and postchange groups (11.0 [IQR: 6.0–19.0] days vs. 9.0 [IQR: 4.0–15.0] days, *P* = 0.02). Similarly, there was no significant difference in hospital stay between the prechange and postchange groups (40.0 [IQR: 24.0–69.0] days vs. 44.0 [IQR: 22.0–73.0] days, *P* = 0.77) (Table 3).

## 4. Discussion

In this retrospective before-and-after study, we examined the differences in ICU mortality and other outcomes between septic patients who had undergone mechanical ventilation with conventional oxygenation targets and those who had undergone mechanical ventilation with permissive hypoxia and hyperoxia avoidance. Although the latter approach did not significantly improve patient survival, it was associated with reductions in mechanical ventilation duration and ICU stay. Accordingly, permissive hypoxia and hyperoxia avoidance may help to increase patient turnover and access to ventilators. This optimization of resource utilization in the ICU is crucial for situations where ventilators and ICU beds are in low supply.

The Intensive Care Unit Randomized Trial Comparing Two Approaches to Oxygen Therapy (ICU-ROX) was conducted in Australia and New Zealand to compare a conservative-oxygen group (if SpO_2_ reached 97%, FiO_2_ was lowered until it reached 0.21 or SpO_2_ returned to an acceptable level) and a usual-oxygen group (SpO_2_ had no protocol-defined upper limit, but had a targeted lower limit of 90%) [16]. That study found no significant difference in the number of ventilator-free days between the groups. While our study focused on septic patients, ICU-ROX included nonseptic patients with elective surgeries and patients admitted to emergency departments. Furthermore, the conservative-oxygen group in ICU-ROX had a median PaO_2_ of 110 mmHg, whereas our study's hyperoxia avoidance (postchange) group kept PaO_2_ levels below 110 mmHg. Our observed reduction in mechanical ventilation duration in the postchange group may have been influenced by this hyperoxia avoidance strategy.

Other trials are also underway to investigate the potential benefits of conservative ventilatory strategies. The Targeted OXygen therapY in Critical illness (TOXYC) study is a multicenter randomized controlled trial being conducted in the UK to compare the effects of SpO_2_ targets of 88–92% and ≥96% on outcomes in mechanically ventilated patients with respiratory failure [4]. Similarly, the Handling Oxygenation Targets in the Intensive Care Unit (HOT-ICU) trial in Denmark is comparing the effects of PaO_2_ targets of 8 kPa (60 mmHg) and 12 kPa (90 mmHg) on 90-day mortality in adults with hypoxemic respiratory failure [17]. In a previous before-and-after study of 105 adults who required mechanical ventilation for more than 48 hours at an Australian tertiary care hospital, it was found that an SpO_2_ target of 90–92% was associated with significant risk reductions for new nonrespiratory organ failure and 28-day mortality when compared with conventional oxygen therapy [18]. In contrast, an Australian multicenter randomized controlled trial found no significant differences in ICU mortality or 90-day mortality between SpO_2_ targets of 88–92% and ≥96%. Although some studies did not detect any significant differences in outcomes between permissive hypoxia and conventional oxygenation strategies [19, 20], one study reported an association between permissive hypoxia and reduced mortality [21]. Another study found no significant associations between conservative oxygen therapy and reductions in mortality or hospital stay in septic patients [22], which corroborates our findings. A retrospective cohort study conducted in the Netherlands reported that neither FiO_2_ nor positive end-expiratory pressure settings were reduced in 78% of mechanically ventilated ICU patients with PaO_2_ exceeding 120 mmHg for FiO_2_ targets of 0.21–0.4 [7]. We posit that permissive hypoxia and hyperoxia avoidance may contribute to the reduction of mechanical ventilation duration and ICU stay due to the continuous and aggressive management in response to each patient's oxygenation levels.

The use of high-concentration oxygen therapy is associated with pulmonary cellular damage and decreased mucus clearance, which can depress the immune system and elevate the risk of pneumonia [23]. In particular, severe lung injury occurs more easily for PaO_2_ of 450 mmHg or more and FiO_2_ of 0.6 or more [23]. Other studies have also reported that hyperoxia after nontraumatic cardiac arrest or stroke is associated with increased mortality [24, 25]. An analysis of immunocompromised patients found that high-concentration oxygen therapy was significantly associated with increased complications, but not with mortality [26]. It should be noted that our hospital's policy does not dictate strict oxygenation control in which targets must be met at all times. Instead, it requires that physicians adjust the oxygen fractions and/or ventilatory mode settings after recognizing the occurrence of unsupportable hypoxia and hyperoxia. We found no signs of adverse events resulting from this policy.

The Acute Respiratory Distress Syndrome Network has proposed a lung-protective ventilation approach [1], and tidal volumes with a plateau pressure of 30 cmH_2_O or less can lead to lower PIP levels. A systematic review reported that patients who had received protective ventilation tended to have shorter mechanical ventilation durations, although this trend was not statistically significant [27]. Our hospital's new ventilation policy was designed to control PaO_2_ levels through PIP settings, but did not seek to reduce PIP as a target. Nevertheless, this could have contributed to the shorter mechanical ventilation duration observed in the postchange group. Our analysis may therefore also show the possible effects of a lung-protective ventilation approach on reducing mechanical ventilation duration.

As our ICU uses APRV for all patients with respiratory failure, this study is characterized by its focus on APRV-treated cases. Extracorporeal membrane oxygenation was not used in our patients during the study period. Previous studies have shown that APRV allows the continuous and rapid improvement of oxygenation in ARDS patients [26, 28, 29]. The use of APRV in our respiratory failure patients (including ARDS patients) may have contributed to improvements in oxygenation and warrants further investigation. In addition, neuromuscular blocking drugs were only used in 3 emergent intubation cases (prechange: 1, postchange: 2). Prone positioning was performed for all patients in both groups by nursing staff. However, prone positioning for more than 12 hours can place a heavy workload on medical staff and was not utilized in cases where patient safety could not be ensured. In this way, there were no prominent intergroup differences in the use of therapies that could potentially affect respiratory failure rates.

Our analysis detected a significant difference in SOFA score between the 2 groups, which is likely due to the higher SOFA cardiovascular score in the postchange group. While sepsis is characterized by the presence of organ dysfunction, the identification of septic shock requires measured variables such as blood pressure and lactate levels [14]. However, our dataset lacked this information, and we were unable to identify septic shock cases in our sample. Consequently, we could not determine if the postchange group had a higher proportion of septic shock cases than the prechange group. Nevertheless, the multivariate analysis adjusted for the differences in SOFA score, and it is therefore likely that the results are indicative of the effects of the ventilation policy change.

Our findings should be considered in the context of several limitations. First, this was a retrospective single-center study with a relatively small sample size, and our results may lack generalizability. Second, the before-and-after design of this study may have introduced biases such as observer bias or history bias. To reduce these potential biases and improve the validity of our findings, we employed multivariate logistic regression analyses and propensity score matching to account for variations in baseline characteristics. However, multicenter prospective studies are needed to confirm or refute our findings. Third, there may be confounding factors that were not included in analysis. For example, the analysis did not account for differences in underlying disease. Severe diseases such as chronic heart failure, obstructive and/or restrictive pulmonary disease, and chronic kidney failure would affect patient prognosis. In addition, the management of critically ill patients (e.g., early mobilization and early enteral nutrition) may have improved over time, and it is possible that this and other unidentified confounding factors had influenced the reductions in mechanical ventilation duration and ICU stay during the relatively long study period. Fourth, we did not use any other ventilatory mode apart from APRV. Therefore, our results may have generalizability issues for other ventilatory modes. Fifth, we could not explain why reductions in mechanical ventilation duration and ICU stay did not lead to corresponding reductions in hospital stay. In Japan, acute care hospitals also fulfill the roles that would be assumed by subacute care hospitals in other countries. Therefore, these longer hospitalization durations may have been less affected by the shorter ICU stay. Finally, a post hoc power analysis for ICU mortality showed that our study had a statistical power of 0.27, indicating a risk of type II error. In order to obtain a statistical power of 0.8, each group requires a minimum of 462 cases. Accordingly, there is a need for further analyses using larger sample sizes to verify or refute our findings.

In conclusion, the policy of permissive hypoxia and hyperoxia avoidance for septic patients requiring mechanical ventilation did not significantly improve prognosis. However, the shift in oxygenation targets was associated with reductions in mechanical ventilation duration and ICU stay, which would increase access to ventilators and other resources in the ICU. Nevertheless, further studies using larger study populations and multiple institutions are needed to verify our findings.

## Figures and Tables

**Table 1:** Patient characteristics in the prechange and postchange groups (*n* = 213).

Variables	Prechange group (*n* = 83)	Postchange group (*n* = 130)	*P* value
Age (years)	67.7 ± 12.9	68.9 ± 13.4	0.52
Male (%)	69.9	63.1	0.31
Height (cm)	160.6 ± 8.2	160.0 ± 8.9	0.61
Body weight (kg)	58.3 ± 15.5	55.1 ± 11.6	0.09
BMI (kg/m^2^)	22.6 ± 5.4	21.5 ± 3.9	0.10
SOFA score (IQR)	8.0 (6.0-10.3)	9.0 (7.0-12.0)	0.03
SOFA respiratory score	2.0 (1.0-3.0)	2.0 (1.0-3.0)	0.66
SOFA cardiovascular score	1.0 (0-3.0)	3.0 (0.5-3.0)	0.001
SOFA liver score	0 (0-1.0)	0 (0-1.0)	0.40
SOFA renal score	0 (0-2.0)	1.0 (0-2.0)	0.60
SOFA coagulation score	2.0 (1.0-3.0)	1.0 (0-2.0)	0.72
SOFA neurologic score	3.0 (1.0-4.0)	2.0 (1.0-4.0)	0.82
ARDS severity (%)			
Mild	3.6	1.5	0.16
Moderate	4.8	5.4	
Severe	9.6	14.6	
Source of sepsis (%)			
Lung	48.2	37.7	0.40
Abdomen	33.7	41.5	
Urinary tract	7.2	4.6	
Soft tissue	3.6	10.0	
Mediastinum	1.2	0	
Endocardium	1.2	3.1	
Others	4.8	3.1	

Values are presented as mean ± standard deviation for continuous variables and percentage or score (IQR) for categorical variables. Abbreviations: BMI: body mass index; SOFA: Sequential Organ Failure Assessment; ARDS: acute respiratory distress syndrome; IQR: interquartile range.

**Table 2:** Patient characteristics in the prechange and postchange groups after propensity score matching (*n* = 142).

Variables	Prechange group (*n* = 71)	Postchange group (*n* = 71)	*P* value
Age (years)	67.8 ± 12.7	67.1 ± 13.4	0.76
Male (%)	69.0	64.8	0.59
Height (cm)	160.6 ± 8.5	160.6 ± 8.8	0.95
Body weight (kg)	58.7 ± 16.1	55.9 ± 12.1	0.25
BMI (kg/m^2^)	22.7 ± 5.5	21.6 ± 4.2	0.19
SOFA score (IQR)	8.0 (6.0-10.0)	8.0 (6.0-10.0)	0.75
SOFA respiratory score	2.0 (1.0-3.0)	2.0 (1.0-3.0)	0.91
SOFA cardiovascular score	1.0 (0-3.0)	1.0 (0-3.0)	0.79
SOFA liver score	0 (0-1.0)	0 (0-1.0)	0.41
SOFA renal score	0 (0-2.0)	1.0 (0-2.0)	0.85
SOFA coagulation score	1.0 (0-2.0)	1.0 (0-2.0)	0.97
SOFA neurologic score	3.0 (1.0-4.0)	2.0 (1.0-3.0)	0.55
ARDS severity (%)			
Mild	4.2	1.4	0.36
Moderate	2.8	7.0	
Severe	11.3	15.5	
Source of sepsis (%)			
Lung	49.3	36.6	0.35
Abdomen	29.6	39.4	
Urinary tract	8.5	8.5	
Soft tissue	4.2	11.3	
Mediastinum	1.4	0	
Endocardium	1.4	1.4	
Others	5.6	2.8	

Values are presented as mean ± standard deviation for continuous variables and percentage or score (IQR) for categorical variables. Abbreviations: BMI: body mass index; SOFA: Sequential Organ Failure Assessment; ARDS: acute respiratory distress syndrome; IQR: interquartile range.

**Table 3:** Patient outcomes in the prechange and postchange groups (*n* = 213).

Variables	Prechange group (*n* = 83)	Postchange group (*n* = 130)	*P* value
PIP (IQR) (cmH_2_O)			
Just after tracheal intubation	25.0 (20.0-27.0)	20.0 (20.0-25.0)	<0.001
Max PIP within 48 hours	25.0 (25.0-30.0)	23.0 (20.0-25.0)	<0.001
Plateau pressure (IQR) (cmH_2_O)			
Just after tracheal intubation	23.0 (14.0-24.0)	16.0 (11.0-23.0)	<0.001
At max PIP within 48 hours	23.0 (23.0-28.0)	19.0 (13.0-24.0)	<0.001
P/F ratio (IQR)			
Just before tracheal intubation	154.0 (89.2-279.9)	183.3 (91.0-369.4)	0.19
Just after tracheal intubation	227.0 (153.0-313.9)	237.0 (149.5-349.2)	0.58
At max PIP within 48 hours	250.0 (158.3-385.4)	243.3 (148.4-340.0)	0.38
Barotrauma (%)	12.0	11.5	0.91
Duration of MV (IQR) (days)	11.0 (6.0-19.0)	7.0 (3.0-14.0)	0.01
ICU stay (IQR) (days)	11.0 (6.0-19.0)	9.0 (4.0-15.0)	0.02
Hospital stay (IQR) (days)	40.0 (24.0-69.0)	44.0 (22.0-73.0)	0.77
ICU mortality (%)	31.3	23.1	0.18
In-hospital mortality (%)	44.6	44.6	1.00

Values are presented as median (IQR) for continuous variables and percentage for categorical variables. Abbreviations: PIP: peak inspiratory pressure; IQR: interquartile range; P/F: partial pressure of arterial oxygen/fraction of inspiratory oxygen; MV: mechanical ventilation.

**Table 4:** Results of the logistic regression analysis of ICU mortality (*n* = 213).

	Odds ratio	95% CI	*P* value
Unadjusted			
Postchange group (ref. prechange group)	0.70	0.22-2.23	0.55
Adjusted			
Postchange group (ref. prechange group)	0.67	0.33-1.43	0.31
Age	0.98	0.95-1.00	0.09
Male (ref. female)	1.04	0.46-2.35	0.93
BMI	1.01	0.93-1.09	0.81
Source of sepsis (ref. abdomen)			
Lung	1.04	0.42-2.56	0.93
Urinary tract	0.64	0.12-3.46	0.61
Soft tissue	0.33	0.04-2.98	0.32
Mediastinum	—	—	—
Endocardium	2.75	0.36-21.0	0.33
Others	8.72	1.15-66.2	0.04
SOFA score	1.15	1.02-1.29	0.02
P/F ratio just before tracheal intubation	0.99	0.99-1.00	0.04

Abbreviations: ICU: intensive care unit; CI: confidence interval; BMI: body mass index; SOFA: Sequential Organ Failure Assessment; P/F: partial pressure of arterial oxygen/fraction of inspiratory oxygen.

**Table 5:** Results of the logistic regression analysis of ICU mortality in propensity score-matched patients (*n* = 142).

	Odds ratio	95% CI	*P* value
Unadjusted			
Postchange group (ref. prechange group)	0.73	0.34-1.59	0.43
Adjusted			
Postchange group (ref. prechange group)	0.81	0.32-2.04	0.66
Age	0.96	0.92-0.99	0.02
Male (ref. female)	1.02	0.34-3.05	0.98
BMI	1.02	0.93-1.12	0.67
Source of sepsis (ref. abdomen)			
Lung	2.73	0.70-10.67	0.15
Urinary tract	1.38	0.20-9.39	0.74
Soft tissue	1.19	0.10-14.51	0.89
Mediastinum	—	—	—
Endocardium	5.78	0.24-138.72	0.28
Others	14.61	1.40-152.87	0.03
SOFA score	1.18	1.01-1.38	0.04
P/F ratio just before tracheal intubation	0.99	0.99-1.00	0.47

Abbreviations: ICU: intensive care unit; CI: confidence interval; BMI: body mass index; SOFA: Sequential Organ Failure Assessment; P/F: partial pressure of arterial oxygen/fraction of inspiratory oxygen.

**Table 6:** Results of the logistic regression analysis of ICU mortality in ARDS patients (*n* = 50).

	Odds ratio	95% CI	*P* value
Unadjusted			
Postchange group (ref. prechange group)	0.66	0.36-1.22	0.18
Adjusted			
Postchange group (ref. prechange group)	0.65	0.33-1.31	0.23
SOFA score	1.15	1.03-1.28	0.01
P/F ratio just before tracheal intubation	0.99	0.99-1.00	0.01

Abbreviations: ICU: intensive care unit; ARDS: acute respiratory distress syndrome; CI: confidence interval; SOFA: Sequential Organ Failure Assessment; P/F: partial pressure of arterial oxygen/fraction of inspiratory oxygen.

## Data Availability

The dataset used in this study is available from the corresponding author on reasonable request.
